# Bioturbation in a Declining Oxygen Environment, *in situ* Observations from Wormcam

**DOI:** 10.1371/journal.pone.0034539

**Published:** 2012-04-06

**Authors:** S. Kersey Sturdivant, Robert J. Díaz, George R. Cutter

**Affiliations:** 1 Virginia Institute of Marine Science, College of William & Mary, Gloucester Pt., Virginia, United States of America; 2 National Oceanic and Atmospheric Administration, Cordell Bank National Marine Sanctuaries Program, Point Reyes Station, California, United States of America; 3 National Oceanic and Atmospheric Administration, Southwest Fisheries Science Center, La Jolla, California, United States of America; Swansea University, United Kingdom

## Abstract

Bioturbation, the displacement and mixing of sediment particles by fauna or flora, facilitates life supporting processes by increasing the quality of marine sediments. In the marine environment bioturbation is primarily mediated by infaunal organisms, which are susceptible to perturbations in their surrounding environment due to their sedentary life history traits. Of particular concern is hypoxia, dissolved oxygen (DO) concentrations ≤2.8 mg l^−1^, a prevalent and persistent problem that affects both pelagic and benthic fauna. A benthic observing system (Wormcam) consisting of a buoy, telemetering electronics, sediment profile camera, and water quality datasonde was developed and deployed in the Rappahannock River, VA, USA, in an area known to experience seasonal hypoxia from early spring to late fall. Wormcam transmitted a time series of *in situ* images and water quality data, to a website via wireless internet modem, for 5 months spanning normoxic and hypoxic periods. Hypoxia was found to significantly reduce bioturbation through reductions in burrow lengths, burrow production, and burrowing depth. Although infaunal activity was greatly reduced during hypoxic and near anoxic conditions, some individuals remained active. Low concentrations of DO in the water column limited bioturbation by infaunal burrowers and likely reduced redox cycling between aerobic and anaerobic states. This study emphasizes the importance of *in situ* observations for understanding how components of an ecosystem respond to hypoxia.

## Introduction

Bioturbation describes the biological reworking of sediments by flora, fauna, or microbial activity [1]. This study focuses on infaunal bioturbation, as it has been shown to play a vital role in regulating marine sediment geochemical and physical properties [2], [3] as well as affecting ecosystem function [1]. Sediment permeability, chemical gradients in pore water, remineralization, and inorganic nutrient efflux are a few of the sediment properties and functions regulated by infaunal bioturbation [4].

The sessile nature of the infauna makes them susceptible to changes in the surrounding environment. Consequently, any factors that influence their behavior can affect bioturbation. One of the most important is hypoxia, dissolved oxygen (DO) concentrations of ≤2.8 mg l^−1^ [5], an emergent threat to coastal marine systems worldwide [6]. Documented cases of coastal hypoxia have increased exponentially since the 1960s, exacerbated by anthropogenic eutrophication, fueled by riverine runoff of fertilizers and the burning of fossil fuels [6]. This enhances primary production in coastal waters, resulting in an accumulation of particulate organic matter and high microbial activity that consumes DO in bottoms waters [7]. Hypoxia has been shown to influence the behavior of infauna [7] and eventually cause mortality from prolonged exposure [8]. Hypoxia also affects sediment geochemistry, resulting in more reduced conditions and a shallowing of the redox-potential discontinuity (RPD) layer [9]. Results from laboratory and field studies suggest that infaunal bioturbation is severely reduced, if not stagnant during periods of hypoxia [10], [11].

Laboratory studies are effective in providing insight into unknown processes, but can only simulate the complexities observed *in situ* [12]. The development of sediment profile cameras has enabled *in situ* observations of organism-sediment interactions [13]. Diaz and Cutter [14] and Solan and Kennedy [15] used time-lapse profile cameras to document *in situ* burrowing and formation of other biogenic structures. We developed Wormcam as an *in situ* benthic observing system that is a combination of a sediment profile camera and datasonde to collect a time-lapse series of 2-dimensional vertical cross-section images and water quality data. Information collected was transmitted in near real-time, every 60 minutes using a wireless internet router to our website. Our objectives were to assess, via *in situ* observations, the impacts of hypoxia on bioturbation, infaunal behavior, and sediment geochemistry. Our study site was located in the Rappahannock River, Virginia, which is known to experience episodes of summertime hypoxia that cause avoidance by mobile fauna and mortality of infauna [16]. Hypoxia was observed to persist in bottom waters of the lower Rappahannock throughout the summer, with sustained hypoxia in the deep portions and periodic reoxygenation of bottom water in the shallower regions. These reoxygenation events are closely related to spring tide mixing [17].

## Results

**Figure 1:**
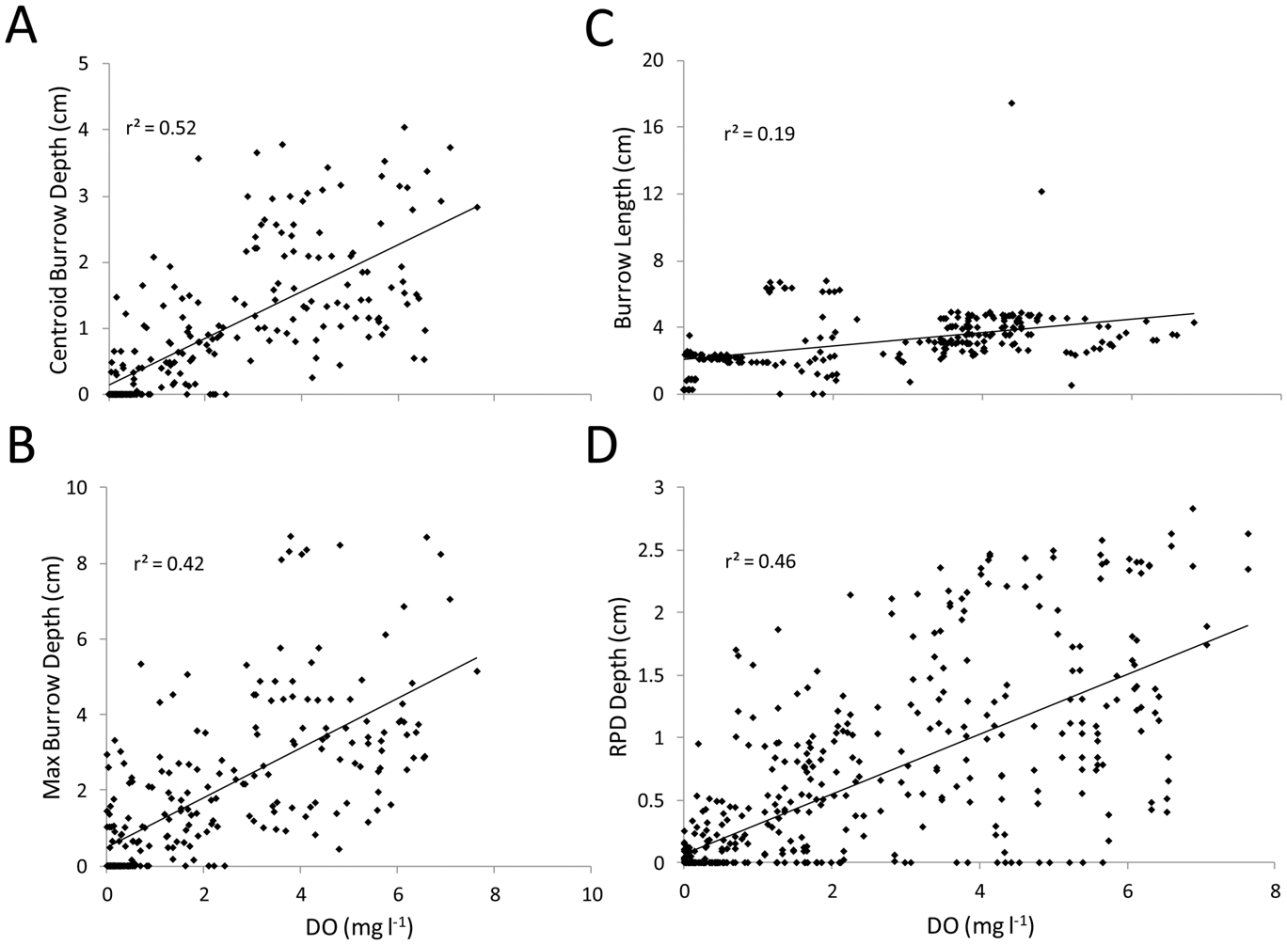
DO concentration's relationship with bioturbation parameters. Relationship of DO concentrations and centroid (A), maximum burrow depths (B), burrow length (C), and aRPD depth (D). Significant positive relationships were found for centroid (p<0.0005, F = 254.48) and maximum (p<0.0005, F = 191.37) burrow depths as well as for burrow length (p<0.0005, F = 95.32) and RPD depth (p<0.0005, F = 399.98).

**Figure 2:**
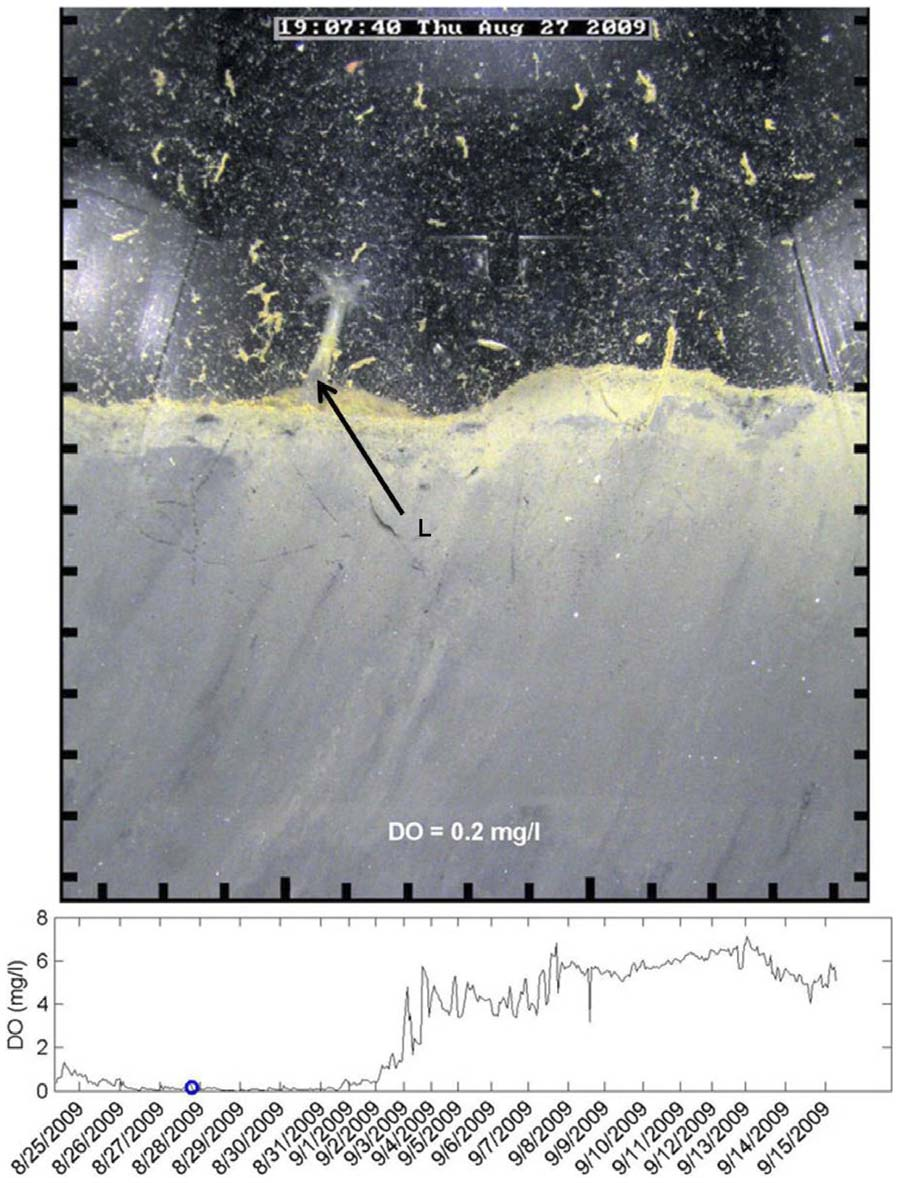
Hypoxia's impact on infauna behavior. The holothurian, *Leptosynapta tenuis* (L), observed extending out of the sediment during near anoxic conditions. Scale around image is in cm units, and the blue circle on the graph shows the DO concentration for the image. Light artifacts from reflection in the prism are visible on the edge of the image.

Over the 5-month study, bottom-water temperature ranged from 18 to 29°C and salinity from 12 to 19 psu. Burrowing activity was detected within an hour post-deployment of Wormcam; small tubificid-like worms were observed first during normoxic conditions and small spionid-like worms during hypoxia. These were the dominant taxa in corresponding sediment grabs collected <10 m from Wormcam (Table 1). As DO concentrations declined the centroid and maximum depth of infaunal burrows became shallower (Fig. 1a–b), to the point where organisms were seen extending their bodies above the sediment surface during prolonged periods of low DO (Fig. 2). Mean maximum burrow depth was 5.6 cm (SD = 2.0) in the first period where normoxia transitioned to hypoxia, 1.1 cm (SD = 1.3) in the prolonged period of hypoxia and anoxia, and 2.7 cm (SD = 1.1) in the subsequent rebound to normoxia.

**Table 1:** Abundance of species collected in grabs (0.04 m^2^) at Wormcam site by date.

Taxa	05/07/2009	06/02/2009	06/09/2009	07/21/2009	08/03/2009	08/24/2009
*Neanthes succinea*	1	0	1	0	1	0
*Paraprionospio pinnata*	7	3	7	1	1	3
*Mediomastus ambiseta*	5	1	3	0	0	0
*Sigambra tentaculata*	1	0	1	0	0	0
*Tubificoidies spp.*	7	0	0	0	0	0
*Amphipoda (unknown)*	1	0	1	0	0	0
*Leitoscoplos robustus*	1	0	0	0	0	0
*Streblospio benedicti*	2	2	0	0	0	0
*Eteone heteropoda*	0	1	0	0	0	0
*Glycinde solataria*	0	0	1	0	0	0
*Acteocina caniliculata*	0	0	1	0	0	0
Sea anemone	0	0	0	0	1	0

On average the initial burrow lengths were ≥70% of the maximum length, indicating that the majority of burrow formation was completed within an hour–the time interval between images. Initial length of some burrows was >90% of the maximum length. A significant positive relationship was found between burrow length and DO concentration (Fig. 1c). Increases in burrow length came primarily from burrows extending into newly accreted sediment; worms would extend burrows back to the surface within the hour during high accretion events but were rarely observed burrowing deeper during erosion events. Of the 15 observed burrows, the majority were destroyed or abandoned due to erosion or biological disturbance; especially from blue crab (*Callinectes sapidus*) and American eel (*Anguilla rostrata*), present during normoxia and presumed to be foraging for prey [18], [19], but neither was observed preying on infauna. During hypoxia, burrows remained in place, but appeared abandoned and lost their oxidized appearance.

**Figure 3:**
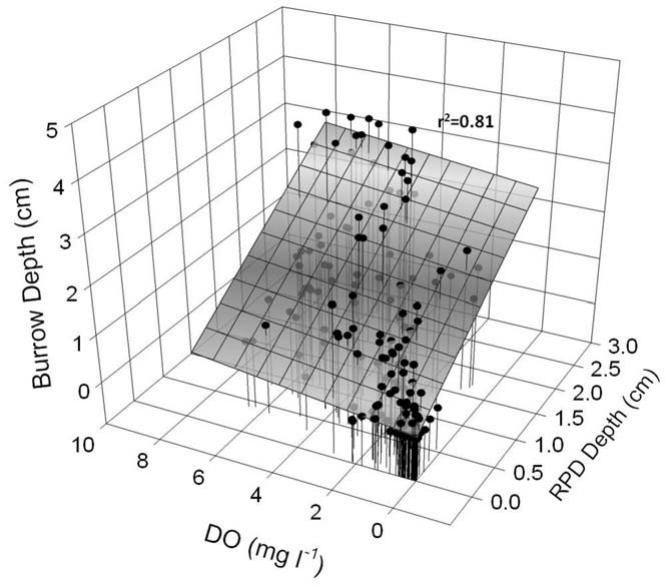
Interaction between DO concentration and apparent-color RPD depth on burrow depth. Relationship of centroid burrow depth with DO concentration and aRPD depth. A significant relationship was found in the interaction between DO and RPD depth on burrow depth (p<0.001, F = 432.35).

Over the study period, burrow production [defined as the change in total burrow length] averaged 0.3 cm h^−1^ (SD = 0.9 cm). Burrow production during normoxia (>2.8 mg O_2_ l^−1^), 0.4 cm h^−1^ (SD = 1.1 cm), was significantly higher (p = 0.001, T = −3.29) than during hypoxia, 0.1 cm h^−1^ (SD = 0.3 cm), by approximately 75%. Burrows generally had two distinct sections, the portion of the burrow above and below the aRPD. On average, burrows extended 2.0 cm (SD = 1.5 cm) below the aRPD. Worms did not appear to favor either side of the aRPD and were observed moving throughout the vertical extent of their burrows during all conditions. During normoxia, the portion of burrows above the aRPD always appeared oxidized (reddish-brown in color), and the portion below the aRPD became oxidized within an hour to an average of 0.1 cm (SD = 0.03 cm) from the burrow wall. During hypoxia, burrows appeared to remain oxidized above the aRPD, but not below. The entire length of burrows appeared completely reduced during periods of severe hypoxia (<0.5 mg l^−1^) and anoxia. The effect of hypoxia on sediment geochemistry was assessed via the depth of the apparent-color RPD (Fig. 1d). As DO concentration decreased the aRPD depth moved closer to the sediment surface and burrow depth significantly declined (Fig. 3), these two parameters accounted for greater than 80% of the observed variability in burrow depth. When anoxia was reached, the aRPD was not discernible.

During periods of severe hypoxia or anoxia, we observed the dynamic nature of bacterial mat formation. As DO declined to 0 mg l^−1^ and anoxic conditions spread to the sediment surface, stringy white sulfur bacteria, likely *Beggiatoa* spp., were observed migrating through the sediment to the surface (Movie S3). Over the 14 day anoxic event (Aug 1–15), bacteria migrated to the sediment surface at 1.2 mm h^−1^ (SD = 3.6 mm), climbed up the window of the housing, and produced copious amounts of organic matter, which then settled onto the sediment surface. The original sediment surface was quickly covered by this unconsolidated mass of bacteria and sediment. Consolidation of this layer occurred beneath the weight of new organic material. By the end of the anoxic period, the sediment surface had risen approximately 7 cm with the top 0.5 cm of the new material being unconsolidated. As DO concentration began to rebound, bacteria migrated en masse, back down into the sediment (Movie S3), and the unconsolidated material left at the sediment surface was eroded by currents within a few hours. A week-long period of normoxia followed and bioturbation was dominated by nereid and capitellid polychaetes. Towards the end of this period, the bacteria started to migrate back to the sediment surface and reformed a bacterial mat over the next hypoxic/anoxic period (Aug 23–September 1).

**Figure 4:**
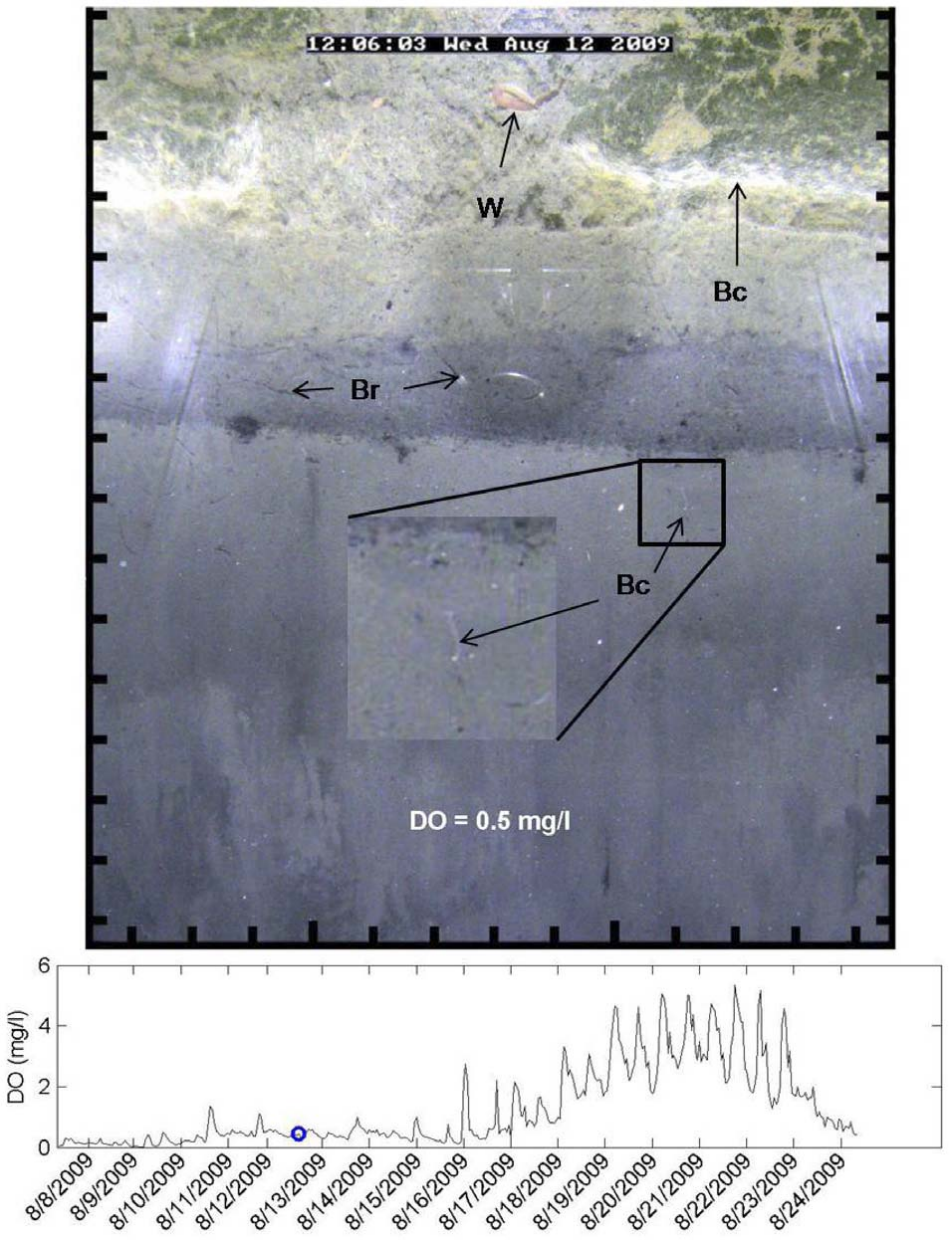
Dynamic nature of the sediment during near anoxic conditions. Sediment profile image showing a worm (W) and worm burrows (Br), during severe hypoxic conditions, and bacteria (Bc) migrating to the sediment surface and producing copious amounts of organic matter. Scale around image is in cm units, and the blue circle on the graph shows the DO concentration for the image. Light artifacts from reflection in the prism are visible on the edge of the image.

**Figure 5:**
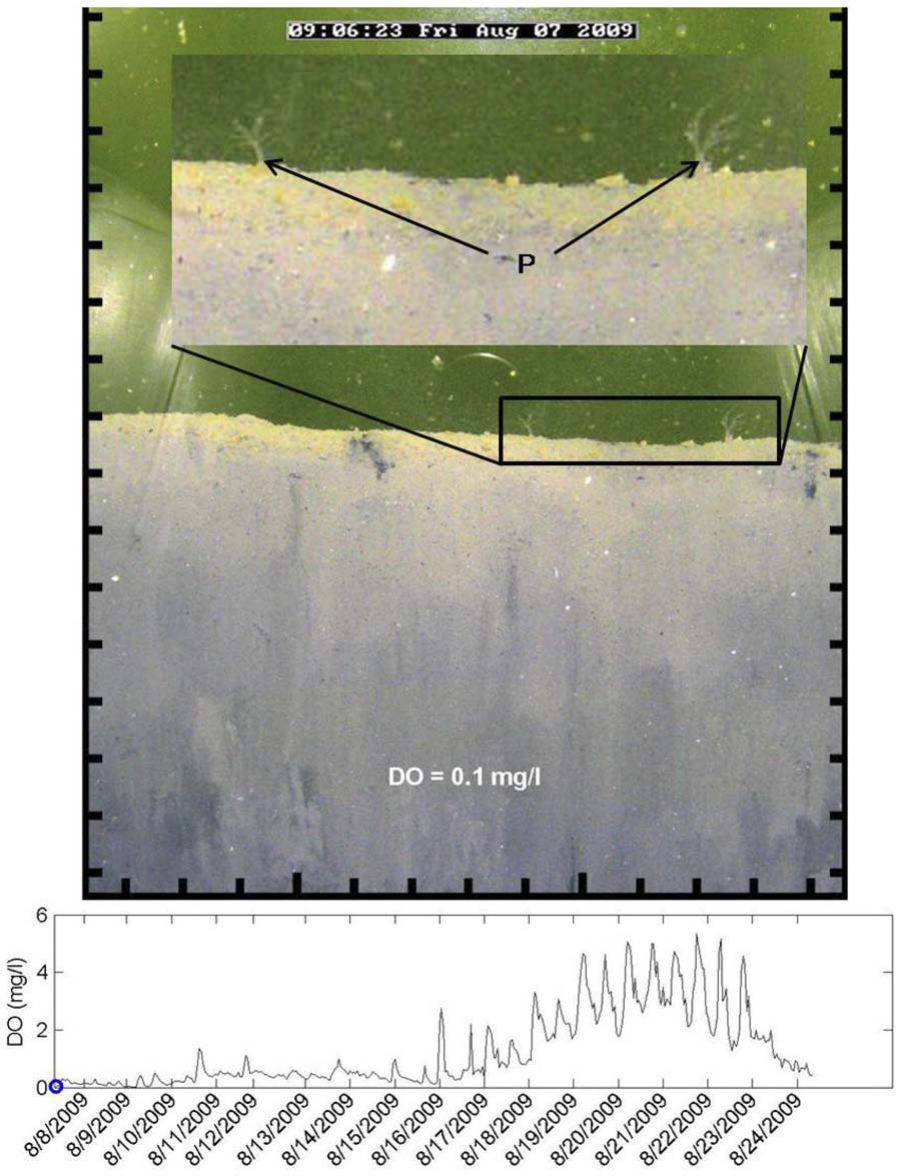
Sediment profile during near anoxic conditions. Sediment profile image showing *Paraprionospio pinnata* (P) at the surface during the onset of a near anoxic event. Scale around image is in cm units, and the blue circle on the graph shows the DO concentration for the image. Light artifacts from reflection in the prism are visible on the edge of the image.

Some infaunal burrowing and movement continued when the water column was severely hypoxic or anoxic (Fig. 4). Prior to the onset of anoxia several spionid polychaetes, *Paraprionospio pinnata*, were observed at the sediment surface with their characteristic palps extended into the water column at a DO of 0.1 mg l^−1^ (Fig. 5). As DO concentration declined further to anoxia it appeared that *P. pinnata* continued to burrow through the sediment and flocculent bacterial mat. Burrows created during this period remained anaerobic and were not inhabited for longer than an hour. This is a conservative estimate, and burrows may have been inhabited for less time, but we only assessed at the one hour sampling interval. Previous *in situ* and laboratory observations of infauna showed increased burrow abandonment and ‘surfacing’ of individuals during hypoxia [10], [11], [14]. At the onset of hypoxia, rather than actively maintain a burrow, Nereid polychaetes were 18 times more likely to abandon burrows and ‘surface’ [14]. Sediment grabs collected during anoxia on 24 August only contained *P. pinnata* (Table 1).

As described in the methods, a burst of 8 to 12 images, 15 seconds apart, were captured every hour; while not used for statistical assessment the burst of images provided interesting behavioral observations in our sediment profile viewing area. For example, we observed worms retracting into burrows upon the presence of a predator, a nereid worm preying on another worm, a goby present during hypoxia, a sea cucumber extending its body and appendages above the sediment-water interface during hypoxia, and apparently a worm using the burrow of another worm (Movie S1, S2, S3, S4).

## Discussion

We found hypoxia to reduce the rates and depth of bioturbation. Burrow depths and lengths were significantly related to DO concentration with shallower burrow depths and reduced burrow lengths during lower oxygen. Reductions in burrow depths and lengths diminished the area of influence of bioturbators, which interferes with ecosystem function by limiting the amount of sediment reworked [15]. The consequences of inhibiting bioturbation are likely to cascade to a variety of physical, biological and chemical processes [20], including organic matter remineralization and decomposition [21], [22], nutrient cycling [23], pollutant release [24], sediment resuspension [25] and microbial activity [26]. The rate of sediment reworking through burrow production was reduced by 75% during hypoxia, under the assumption that the observed organisms were representative of biogenic activity. The burrow formation rate, observed in this study, may be a liberal estimate since burrow formation at the sediment-camera-faceplate interface is facilitated along the interface [27]. But irrespective of the actual burrow formation rate, we would expect low oxygen to exert the same percentage reduction; this provides useful insight into the potential impacts of hypoxia on organism-sediment and sediment-water interactions. Sediment reworking by infuana, through burrow formation, can be classified as either biodiffusive or bioadvective [28]. Biodiffusors randomly mix sediment by free burrowing through the sediments, particularly the small-bodied surface dwelling species, and bioadvectors form more permanently occupied burrows that can penetrate deeper into the sediments and convey sediment upwards or downwards [29], [30], [31]. Both increase sediment permeability [28], hence reductions of the rates of these bioturbation components would result in increased consolidation of sediment, decreasing the area of the sediment-water interface and limiting exchange between the sediment and water column [20], [32].

DO concentrations were found to be positively related to the apparent-color RPD layer depth, with shallower aRPD depths at lower DO concentrations. Early observations of Jørgenson and Fenchel [33] determined that sediments can be divided into oxic, suboxic, and anoxic levels, and the vertical positions of these boundaries vary seasonally and locally in response to the supply of organic matter. Graf [34] found the position of these boundaries to have fundamental consequences for biota, as the anoxic sediment layer, which occurs just below the RPD, tends to be toxic to most animals due to free H_2_S and low pH. With a higher flux in metabolizeable organic matter, sediment oxygen consumption increases along with decreases in bottom oxygenation [35], [36]. The decrease in DO reduces infaunal bioturbation and subsequently sediment ventilation [14], [15]. As a result, the RPD rises within the sediment, thus increasing the likelihood of infaunal exposure to H_2_S [7]. In the anaerobic environment below the RPD, reduced conditions dominate and H_2_S can be present [37]. It is difficult to separate the combined effects of low DO and H_2_S toxicity on marine organisms [38], so to explain the effects that these two factors might have on bioturbation, a multiple regression was performed, and a significant positive relationship was found. The interaction of DO concentration and aRPD depth are both important factors that influenced macrobenthos, limiting bioturbation through a reduction in organism activity and burrowing depth. Reductions of bioturbation affected sediment geochemistry via reduced oxygen diffusion across burrow walls. Reducing DO concentration below the sediment surface would lead to reduced oscillation between oxic and anoxic conditions. This cyclical redox oscillation is common within individual burrow structures and is accompanied by rapid switching in dominant metabolic processes [22]. During normoxic conditions, oxygen appeared to diffuse an average of 0.1 cm (SD = 0.03 cm) from burrow walls below the aRPD and facilitate redox oscillation. This oxic layer was not discernible around burrow walls below the aRPD during hypoxia facilitating a more uniform state of anaerobic decomposition [22]. Diaz and Cutter [14] observed worm activity to correspond with increased oxygen diffusion across burrow walls below the aRPD during normoxia.

*In situ* Wormcam images revealed the dynamic nature of the benthic environment and quantified the relationship between DO concentration and infaunal bioturbation. Over a 14-day period of anoxia (Aug 1–15), filamentous bacteria were observed migrating through the sediment and producing a flocculent mat on the Wormcam faceplate and the sediment surface. Microbial migration and formation of microbial mats has been documented [9], [39], [40], however, the observation of this process and the subsequent burrowing of worms throughout the sediment and bacterial mat are new. We also found some portion of the infauna to remain active during hypoxia and even anoxia. Infaunal activity was observed during anoxia and in the presence of sulfur-oxidizing bacteria [41] from the sediment surface to 5 cm below the surface. Riedel et al. [42] *in situ* observations of surface-faunal behavior during hypoxia also found infauna to surface. However, laboratory experimental data would predict mortality and no infaunal activity during anoxia [8]. Although active worm burrowing was observed, the burrows created during this period remained anoxic, indicating that bioturbation could also act as a process to aid the diffusion of anaerobic compounds out of the sediments and into the water column.

The burrows observed during severe hypoxia were likely created by the spionid polychaete *Paraprionospio pinnata*, collected in corresponding benthic grabs and images. *P. pinnata* is a highly adaptive species implementing both suspension and deposit feeding strategies and respiratory morphology and physiology that supports survival in low DO [43]. This plasticity, in the traits of *P. pinnata*, may explain their activity during prolonged hypoxic/anoxic events. Skipper et al. [44] defines plasticity as ‘the capacity of organisms or cells to alter their phenotype in response to changes in their environment.’ Before the onset of anoxia, on multiple occasions the DO concentrations at our site became hypoxic for a short duration. These low DO events may have pre-conditioned the infauna physiologically for the subsequent near-anoxic event. Childress and Siebel [45] discuss three methods organisms use to cope with low oxygen: increasing oxygen uptake, decreasing metabolic demands, or utilizing anaerobic metabolism. In response to the infrequent short-duration low oxygen events, worms not killed would have a physiological response to produce more haemoglobin; increasing the capacity of their coelomic fluid to uptake oxygen and subsequently the ability to cope with the next low oxygen event [46], [47]. *P. pinnata* are also morphologically well adapted to deal with a low oxygen environment having elongated, proliferated and numerous branchia [43], [48]. We could not determine from the images if *P. pinnata* decreased their metabolic demand, but *P. pinnata* observed during near-anoxia were highly active. Gonzalez and Quiñones [49] showed that *P. pinnata* possess all four subsets of pyruvate oxidoreductases (LDH, ALPDH, OPPDH, and STRDH), which are enzymatic adaptations associated with anaerobic metabolism during low DO. Levin [50] suggests that the high numbers and variety of these enzymes may ‘confer metabolic plasticity, and could explain the success of *P. pinnata* in hypoxic settings around the world’. It is also possible that association of *P. pinnata* with the mat-forming, sulfide-oxidizing bacteria, observed in the images, placed them in less sulphidic microhabitats [51]. The reduction of a toxic environment, that would normally be coupled with low DO, may have relieved enough stress allowing *P. pinnata* to survive; the detrimental impact of multiple-stressors is thoroughly documented [52].

Hypoxia reduced bioturbation through significant reductions in burrow lengths, burrow production, and burrow depth. Although some infaunal activity was observed during hypoxic and anoxic conditions, the low concentrations of DO and reduced burrowing limited diffusion of oxygen into the sediment, leading to a corresponding reduction in redox cycling and more uniform anaerobic conditions. *In situ* observations from Wormcam showed that some worms remained active during hypoxia, reflecting their physiological plasticity and perhaps an ability to capitalize on the newly available bacterial organic material. Thus a fraction of infaunal bioturbation continues during low DO.

For seabed regions of particular concern, or those being monitored by benthic sampling activities, this type of observation system could contribute to a better understanding of those processes. Typical monitoring relies on data collection of samples or images for detailed instantaneous characterization of the system. A continuously-deployed Wormcam, with peripheral sensors, would provide data that relate activity of benthic fauna, on an effectively continuous basis, to water conditions. Hence, scientists and managers would be able to review benthic community responses when telemetered sensor data indicate changes of potential concern, rather than responding with post-event sampling. Increases in monitoring efficiency could then improve biological impact assessments of current (e.g. drilling) and emerging (e.g. renewable energy) marine projects.

## Materials and Methods

### Study Area

The study site was in the mesohaline portion of the Rappahannock River, an estuary of Chesapeake Bay known to experience seasonal hypoxia [17], [53]. Wormcam was deployed approximately 2.5 km northeast of La Grange Creek (Middlesex County), Virginia, based on DO concentrations collected from previous years. Initially Wormcam was deployed at 27 m depth at Location 1 (37°41, 24.8′N, 76°33, 47.9′W), in mid-July it was moved 0.5 km to the east to 32 m at Location 2 (37°41, 25.6′N, 76°33, 37.8′W) to ensure encounter with a prolonged hypoxic event. A benthic bottom permit, #09-0164, was obtained from the Virginia Marine Resources Commission to deploy Wormcam.

### Wormcam

**Figure 6:**
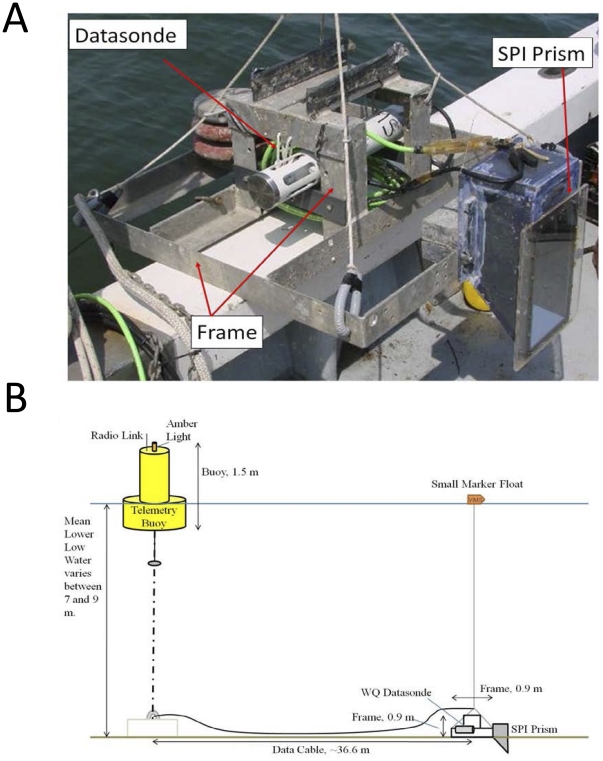
Wormcam apparatus. Image of the Wormcam apparatus (A) and a cross-sectional diagram of the entire Wormcam system (B). Cross-sectional diagram not drawn to scale.

Wormcam consisted of an IQEye model 705 5-megapixel Ethernet camera, placed in a plastic housing with a 45 degree angle at the bottom, which formed a wedge to penetrate into the sediments. A front surface mirror on the back wall in the wedge reflected the sediment profile to the camera. The field of view was 10 cm wide by 15 cm deep. Lighting was provided by a single white LED (Lexeon Star model 5C). The camera was set to take a burst of 8 to 12 images, about 15-seconds apart every hour, that were stored on the camera's memory card. Wormcam was affixed to a low-profile aluminum frame to minimize flow disturbance and to prevent the camera from fully sinking in the sediment (Fig. 6a). Also, the window was extended beyond the edges of the housing to prevent eddy-induced erosion near the corners. A Hach DS500X water-quality datasonde was attached to the frame 20 cm above the sediment and collected DO, salinity, temperature, and depth measurements at one hour intervals synchronous with image capture. The entire system was controlled by a Campbell CR1000 microprocessor and solar-powered from a surface buoy connected by cable to Wormcam (Fig. 6b). A few images and water quality data were transmitted wirelessly to our website via a Sierra Wireless AirLink™ Raven X Ethernet modem in near real-time, which allowed us to track water quality and sediment structure conditions. During maintenance trips the memory card was retrieved and images were downloaded for analysis of biogenic structures and sediment oxidation state; sediment grabs were collected using a Young grab (samples an area of 440 cm^2^ to a depth of 10 cm) and screened through a 0.5 mm sieve to assess benthic community composition.

**Figure 7:**
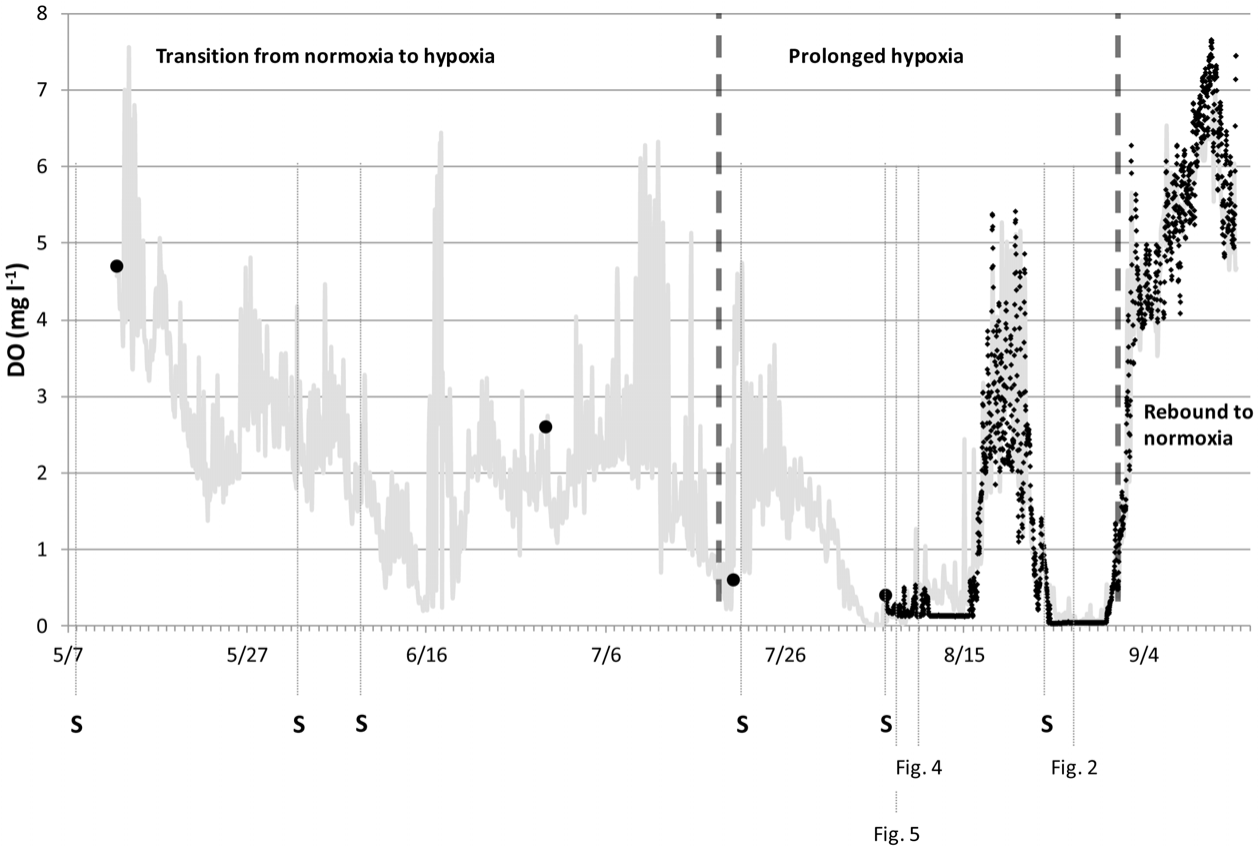
DO record for observation period. DO data from May to September (gray line); black dotted lines separate the three DO periods. Large black dots represent point DO measurements, and small black dots represent DO measurements from the second datasonde. Gray dotted lines denote when sediment grabs (S) were collected and corresponding figures.

Wormcam was deployed for a period of 5 months, from May 13–September 15, 2009, and divided into three oxygen regimes: transition from normoxia to hypoxia (May 13–July 21), prolonged exposure to hypoxia and anoxia (July 21–September 1), and subsequent rebound to normoxia (September 1–September 15, Fig. 7). Maintenance-recoveries and redeployments, a total of six, occurred every 3–4 weeks as needed. During site visits DO measurements were collected from a surface vessel with a handheld YSI Professional Plus water quality meter to verify data from the deployed datasonde. An additional Hach DS500X datasonde was deployed August 7, 2009 until the end of the project to verify DO results. DO concentrations recorded by the Wormcam datasonde were not significantly different from either the handheld YSI (df = 3, T = 0.15, p = 0.888) or the additional datasonde (df = 1790, T = 1.29, p = 0.197), based on paired t-tests.

### Data Analysis

Photoshop (Adobe Systems Inc.) was used to rotate and scale the images, ImageJ (NIH) was used for digital measurements of sediment oxidation state and biogenic structures, and MatLab (The Mathworks) was used to combine images with corresponding DO time-series. Quality control on image data was accomplished by reanalysis of all images by a second analyst. A 6-h interval was used for detecting the effects of hypoxia on visual features and infaunal activities. Oxidation state of the sediment and depth of the apparent-color redox-potential discontinuity (aRPD) was determined by color: reddish-brown sediment was considered oxidized and grayish-black sediment was considered reduced [54]. Mean and maximum burrow depths were recorded as estimates of bioturbation activity from a total of 251 images. The term ‘centroid burrow depth’ was used to describe the geometric mean of all burrow depths in an image and was used to avoid confusion with the term mean in reference to other burrow information; maximum burrow depth was the deepest detectable burrow in an image. The relationships between DO concentration and aRPD, centroid, and max burrow depths were assessed using linear regression.

For each of the three oxygen regimes, a random sample of five burrows was analyzed hourly to determine the effect of DO concentration on burrow length and duration. An hour interval was used for quantifying changes in burrow length and duration from 270 images. For a burrow to be measured, it needed a visible connection to the sediment surface and needed to extend below the aRPD. Natural log transformation was used to achieve normality for burrow length data. Animating the series of images provided information on burrowing activity and fauna behavior relative to DO concentration (Movie S1, S2, S3, S4).

## Supporting Information

Movie S1 **Observations from 15 May to 2 June, 2009.** The DO concentration declines from ∼5.0 mg O_2_ l^−1^ at the start to ∼2.0 mg O_2_ l^−1^ at the end. During this time burrows are created and destroyed, and the sediment surface has net erosion. On multiple occasions you can see the blue crab, *Callinectes sapidus*, and American eel, *Anguilla rostrata*, digging in the sediment. The below table details, though not limited to, some specific behavioral observations. This movie depicts a sequence of images captured every hour, running at a frame rate of 15 hours per second. The black marks around the image are in cm units; date, time, and corresponding DO concentration for the image is indicated at the top and bottom, respectively. The graph below the image displays the DO concentration for the time-span of the movie, and the blue circle bounces around while the movie is played indicating the corresponding DO concentration. Note that this movie is not comprehensive of all the images captured by Wormcam during the time period and if the exhaustive images and movies want to be viewed please feel free to contact the corresponding author. **No. Description. Day; Hour** 1. Blue crab digging in sediment. 22-May; 11:00 2. Blue crab digging in sediment. 30-May; 00:00 3. Worm extending body above sediment surface (right center of image). 24-May; 21:00 4. Mysid shrimp swimming in front of face plate (center of image). 25-May; 05:00 5. Mysid shrimp swimming in front of face plate (center of image). 02-Jun; 06:00 6. Sea cucumber on sediment surface (right center of image). 26-May; 23:00 7. Worm extending body out of burrow (left center of image). 27-May; 00:00 8. American eel digging in sediment. 27-May; 21:00 9. American eel digging in sediment. 29-May; 03:00 10. American eel digging in sediment (right of image). 02-Jun; 05:00 11. Fish on surface of sediment staring at face plate (center of image). 28-May; 09:00.
(M4V)

Movie S2 **Observations from 22 July to 7 August, 2009.** The DO concentration declines from ∼1.5 mg O_2_ l^−1^ at the start to ∼0.1 mg O_2_ l^−1^ at the end. During this time burrows are created and destroyed, and as DO concentration declines burrowing activity decreases. On multiple occasions you can see small fish swimming around during hypoxia in DO concentrations as low as 1.0 mg O_2_ l^−1^. As the DO concentration continues to decline towards the end of the movie, the upper layer of sediment becomes reduced (black in color) and small stringy-white bacteria, likely *Beggiatoa* spp., can be observed migrating through the sediment to the surface. The below table details, though not limited to, some specific behavioral observations. This movie depicts a sequence of images captured every hour, running at a frame rate of 15 hours per second. The black marks around the image are in cm units; date, time, and corresponding DO concentration for the image is indicated at the top and bottom, respectively. The graph below the image displays the DO concentration for the time-span of the movie, and the blue circle bounces around while the movie is played indicating the corresponding DO concentration. Note that this movie is not comprehensive of all the images captured by Wormcam during the time period and if the exhaustive images and movies want to be viewed please feel free to contact the corresponding author. **No. Description. Day; Hour** 1. Worm in u-shaped burrow. 22-Jul; 21:00 2. Worm extending body above sediment surface. 31-Jul; 03:00 3. Small fish on surface of sediment (center of image). 26-Jul; 09:00 4. Small fish on surface of sediment (center of image). 28-Jul; 08:00 5. Small fish swimming around in low DO. 28-Jul; 12:00 6. Small fish swimming around in low DO. 29-Jul; 15:00 7. Full body of a worm on sediment surface. 01-Aug; 15:00.
(M4V)

Move S3 **Observations from 7 August to 24 August, 2009.** The DO concentration is near anoxic at ∼0.1 mg O_2_ l^−1^ at the start and increases to ∼4.0 mg O_2_ l^−1^ by the end of the movie for a number of days only to decline again. During the period of near anoxia, small stringy-white bacteria can be observed migrating to the sediment and producing a bacteria mat. Towards the end of the movie when the DO concentration begins to rebound, these same filamentous-white bacteria, likely *Beggiatoa* spp., can be seen mass-migrating back down into the sediment. A surprising amount of burrowing is observed during the near anoxic conditions in both the consolidated sediment and unconsolidated bacteria mat, though the burrows do not appear to become oxidized. The below table details, though not limited to, some specific behavioral observations. This movie depicts a sequence of images captured every hour, running at a frame rate of 15 hours per second. The black marks around the image are in cm units; date, time, and corresponding DO concentration for the image is indicated at the top and bottom, respectively. The graph below the image displays the DO concentration for the time-span of the movie, and the blue circle bounces around while the movie is played indicating the corresponding DO concentration. Note that this movie is not comprehensive of all the images captured by Wormcam during the time period and if the exhaustive images and movies want to be viewed please feel free to contact the corresponding author. **No. Description. Day; Hour** 1. Worm on the face plate of prism. 08-Aug; 06:00 2. Worm in surface bacteria mat. 12-Aug; 05:00 3. Worm in surface bacteria mat. 12-Aug; 12:00 4. Continued formation of bacteria mat on surface. 12-Aug; 18:00 5. Continued formation of bacteria mat on surface. 14-Aug; 03:00 6. Migration of bacteria back into sediment as DO increases. 16-Aug; 00:00 7. Re-oxygenated sediment. 18-Aug; 15:00.
(M4V)

Movie S4 **Observations from 24 August to 15 September, 2009.** The DO concentration is severely hypoxic at ∼0.6 mg O_2_ l^−1^ at the start and increases to ∼6.0 mg O_2_ l^−1^ by the end of the movie. The below table details, though not limited to, some specific behavioral observations. This movie depicts a sequence of images captured every hour, running at a frame rate of 15 hours per second. The black marks around the image are in cm units; date, time, and corresponding DO concentration for the image is indicated at the top and bottom, respectively. The graph below the image displays the DO concentration for the time-span of the movie, and the blue circle bounces around while the movie is played indicating the corresponding DO concentration. Note that this movie is not comprehensive of all the images captured by Wormcam during the time period and if the exhaustive images and movies want to be viewed please feel free to contact the corresponding author. **No. Description. Day; Hour** 1. Holothurian extending body above sediment surface during low DO. 27-Aug; 19:00 2. Worm on the face plate of prism during low DO. 30-Aug; 00:00 3. Re-oxygenated sediment. 03-Sep; 16:00 4. Goby on the sediment surface. 12-Sep; 16:00 5. Juvenile crab on sediment surface. 14-Sep; 10:00 6. Isopod burrowing into the sediment. 15-Sep; 06:00.
(M4V)
